# OSGIN2 regulates osteogenesis of jawbone BMSCs in osteoporotic rats

**DOI:** 10.1186/s12860-022-00423-8

**Published:** 2022-06-21

**Authors:** Yi Shuai, Bingyao Liu, Liang Rong, Bingyi Shao, Bo Chen, Lei Jin

**Affiliations:** 1 Department of Stomatology, Jinling Hospital, Medical School of Nanjing University, Nanjing, Jiangsu 210002 People’s Republic of China; 2 Department of Stomatology, General Hospital of Eastern Theater Command, PLA, Nanjing, Jiangsu 210002 People’s Republic of China; 3 Department of Endodontics, Affiliated Stomatology Hospital of Chongqing Medical University, Chongqing, 404100 People’s Republic of China

**Keywords:** OSGIN2, Jawbone, Mesenchymal stem cells, Osteoporosis, Oxidative stress

## Abstract

**Background:**

Augmentation of oxidative stress after estrogen deficiency leading to functional deficiency of jawbone bone marrow mesenchymal stem cells (BMSCs) causes jawbone loss in osteoporosis. OSGIN2, an oxidative stress induced factor, has been found to be associated with skeletal diseases. This study aims to investigate the function of OSGIN2 in jawbone BMSCs of osteoporotic rats. Jawbone BMSCs were used.

**Results:**

Oxidative stress was increased in jawbone BMSCs of osteoporotic rats, meanwhile OSGIN2 was also up-regulated. Osteogenesis of jawbone BMSCs was declined under oxidative stress, while silence of OSGIN2 ameliorated the osteogenic deficiency. RORα and its downstream osteogenic markers (BSP and OCN) decreased under oxidative stress, while knocking-down of OSGIN2 restored their expressions. Inhibition of OSGIN2 improved the osteogenesis of jawbone BMSCs under oxidative stress, whereas down-regulation of RORα offset the effect. Intra-jawbone infusion of si-OSGIN2 rescued jawbone loss and promoted new bone deposition of osteoporotic rats.

**Conclusions:**

Oxidative stress is redundant in osteoporosis, which results in up-regulation of OSGIN2. OSGIN2 restricts osteogenic ability of jawbone BMSCs via regulating RORα, while silencing of OSGIN2 rescues the osteogenic deficiency of osteoporotic rats.

**Supplementary Information:**

The online version contains supplementary material available at 10.1186/s12860-022-00423-8.

## Background

Osteoporosis is a relative contraindication of oral implant [1, 2]. Jawbone loss, one of the common accompanied phenotypes of osteoporosis, brings risk to poor osseointegration [3–6]. Jawbone mass is essential for early stability and long-term survival of dental implants [1, 2]. Therefore, concentration on jawbone health of osteoporotic individuals seems to be imperative.

At present, bone compression or local bone grafting is often used to improve the bone density around the implants in osteoporotic patients [7, 8]. However, these methods can only increase the local bone density. Although it can promote initial stability of oral implants, its long-term effect remains unclear during the course of osteoporosis and the reconstruction of jawbone. Thus, treatment of osteoporosis is of great significance to expand the indications of oral implantation and to promote the clinical application. The imbalance between bone formation and bone resorption leads to osteoporotic status [9]. Bone marrow mesenchymal stem cells (BMSCs) play crucial roles in bone formation and regeneration [10]. Osteoporotic bone loss largely ascribes to osteogenic deficiency of BMSCs [11, 12]. Furthermore, therapeutics targeting on BMSCs achieves electrifying outcomes in treatment of osteoporosis [11, 13]. Uncovering the molecular mechanism of BMSCs deficiency is becoming pivotal for management of osteoporosis.

Oxidative stress has been reported to be a key etiology of osteoporosis [14]. Estrogen deficiency and aging result in elevation of reactive oxygen species (ROS), which restricts osteogenic capacity of BMSCs [15, 16]. OSGIN2, a newly reported molecule, is one of the oxidative stress induced growth inhibitor family members, which negatively regulate cell growth responding to redundant oxidative stress (https://www.genecards.org/I). Furthermore, OSGIN2 is associated with Nijmegen Breakage Syndrome, and skeletal disorder has been known as one of its main phenotypes [17]. Additionally, OSGIN2 is highly correlated with the retinitis pigmentosa type 62, which is usually accompanied with bone abnormality (http://www.malacards.org/card/retinitis_pigmentosa_62I). The aforementioned studies suggest the correlation between OSGIN2 and bone metabolism, however the specific mechanism remains elusive.

RORα, a subtype of retinoic acid-related orphan receptors, has been investigated to regulate fate of mesenchymal stem cells, and subsequently lead to osteoporotic diseases [18, 19]. It has been reported that RORα is abundant in BMSCs. Bone sialoprotein (BSP) and osteocalcin (OCN), crucial molecules of skeletal development and mineralization [20–23], have been well reported to facilitate osteogenesis of osteoblasts and mesenchymal stem cells [24–26]. Up-regulation of RORα triggers transcriptions of osteogenic related genes BSP and OCN, and then promotes osteogenic differentiation of BMSCs [18, 27]. After knocking-out RORα, the growth and development of mice bones are significantly depressed, which mainly manifest in the thinning of long bone and the decrease of bone mass [18, 27]. This indicates that RORα has a promotive regulatory effect on the osteogenesis of BMSCs. However, the relationship between OSGIN2 and RORα remains unknown.

In this study, we investigated the mechanism that OSGIN2 responded to oxidative stress and affected osteogenesis of jawbone BMSCs via regulating RORα/BSP-OCN during osteoporosis.

## Results

### Oxidative stress is negatively correlated with osteogenesis of jawbone BMSCs during osteoporosis

**Fig. 1:**
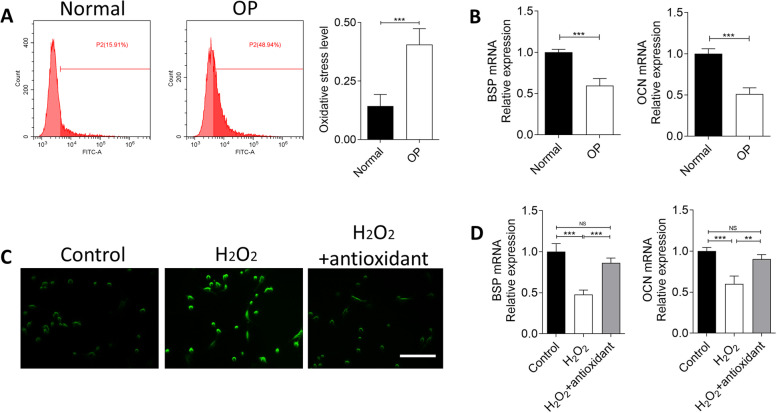
Relationship between oxidative stress and osteogenesis of jawbone BMSCs in osteoporosis. ROS levels were detected using FCM (**A**), and expressions of BSP and OCN mRNAs were detected using qPCR (**B**) between jawbone BMSCs from normal and osteoporotic rats (Normal as control group: non-osteoporosis). ROS levels were detected using fluorescent assay (**C**), and expressions of BSP and OCN mRNAs were detected using qPCR (**D**) in cultured jawbone BMSCs (Control as control group: no H_2_O_2_ or antioxidant treatment). *n* = 5. Scale bar: 200 μm. **p < 0.01, ***p < 0.001, NS: no significance. OP: osteoporosis

To investigate the relationship between oxidative stress and osteogenesis of jawbone BMSCs during osteoporosis, we isolated jawbone BMSCs, tested intracellular ROS and detected osteogenic markers BSP and OCN. Compared to normal BMSCs, BMSCs from osteoporotic jawbones showed significantly higher ROS (Fig. 1A), but declined BSP and OCN expressions (Fig. 1B).

H_2_O_2_ was used to induce ROS generation of jawbone BMSCs to mimic cellular oxidative stress in vitro. After induction, ROS obviously elevated (Fig. 1C), while BSP and OCN decreased (Fig. 1D) compared to control group. However, treatment of catalase restricted the ROS generation (Fig. 1C) and improved BSP and OCN expression (Fig. 1D). All these data indicated that oxidative stress was negatively correlated with osteogenesis of jawbone BMSCs during osteoporosis.

### Expression of OSGIN2 is ascended in jawbone BMSCs during osteoporosis

**Fig. 2:**
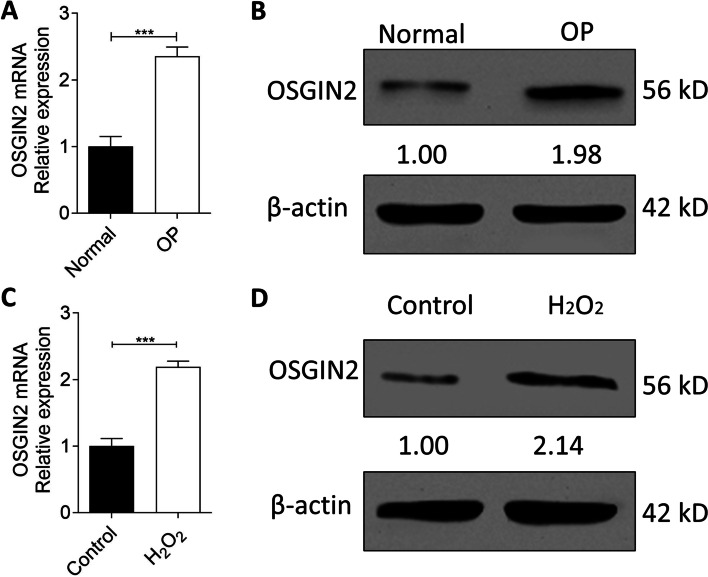
Expression of OSGIN2 in osteoporotic jawbone BMSCs and oxidative stress induced jawbone BMSCs. Expressions of OSGIN2 mRNA (**A**) and protein (**B**) between normal and osteoporotic jawbone BMSCs (Normal as control group: non-osteoporosis). Expressions of OSGIN2 mRNA (**C**) and protein (**D**) between control and H_2_O_2_ induced jawbone BMSCs (Control as control group: no H_2_O_2_ treatment). The blots were cut priorly to hybridisation with antibodies during blotting. *n* = 5. ***p < 0.001. OP: osteoporosis

Compared to normal jawbone BMSCs, expressions of OSGIN2 were sharply increased at both mRNA (Fig. 2A) and protein (Fig. 2B) levels in osteoporotic jawbone BMSCs.

To investigate whether OSGIN2 augmentation was due to increased oxidative stress, OSGIN2 expression in oxidative-stress-induced cellular model was detected. The data exhibited that OSGIN2 was highly increased after induction of oxidative stress (Fig. 2C and D), which implied that oxidative stress triggered the elevation of OSGIN2.

### OSGIN2 negatively regulates osteogenesis of jawbone BMSCs under oxidative stress

**Fig. 3:**
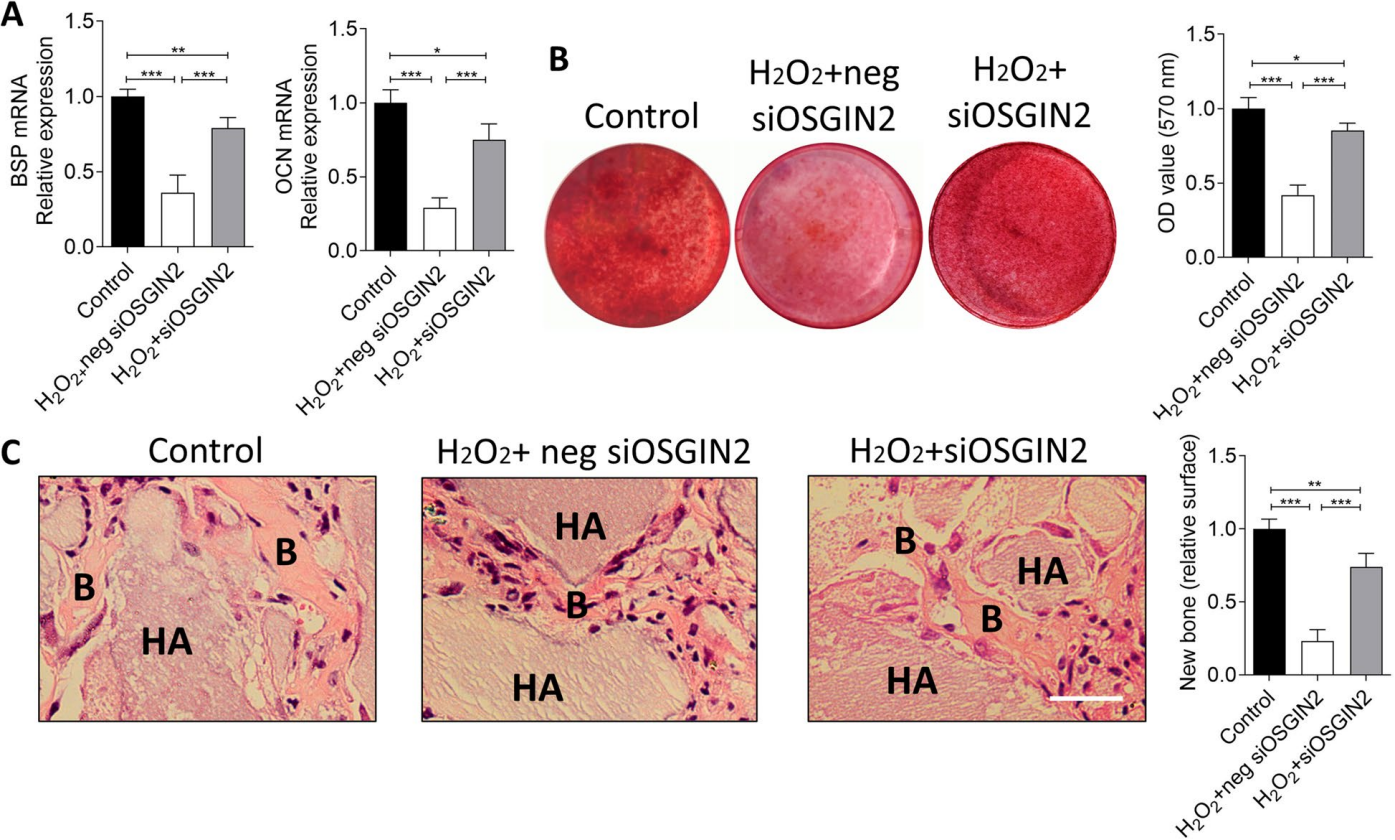
OSGIN2 regulates osteogenesis of jawbone BMSCs under oxidative stress. **A** Expressions of BSP and OCN mRNAs were detected using qPCR, **B** Mineralized nodules were detected using alizarin red, **C** Ectopic bone formations were detected using H&E after treatment of H_2_O_2_ + neg siOSGIN2 and siOSGIN2 (Control as control group: no H_2_O_2_, siOSGIN2 or negative control of siOSGIN2 treatment). *n* = 5. Scale bar: 500 μm. *p < 0.05, **p < 0.01, ***p < 0.001

In order to verify the osteogenesis of jawbone BMSCs under oxidative stress, osteogenic markers, mineralized nodules formation and ectopic bone formation were analyzed after treatment with H_2_O_2_. The data showed that expressions of osteogenic markers BSP and OCN were significantly decreased under oxidative stress, compared to control group (Fig. 3A). In addition, alizarin red assay exhibited that the mineralized nodules formation was declined under oxidative stress, compared to controls (Fig. 3B). Furthermore, the results of ectopic bone formation also displayed similar tendency (Fig. 3C).

Noteworthily, knocking-down of OSGIN2 expression successfully reversed the osteogenic deficiency of jawbone BMSCs caused by oxidative stress, such as the augmentations of the expressions of BSP and OCN (Fig. 3A), mineralized nodules (Fig. 3B) and subcutaneous new bone formation (Fig. 3C). These data suggested that OSGIN2 negatively regulated osteogenesis of jawbone BMSCs under oxidative stress.

### OSGIN2 inhibits jawbone BMSCs osteogenesis under oxidative stress via regulating RORα/BSP-OCN signaling

**Fig. 4:**
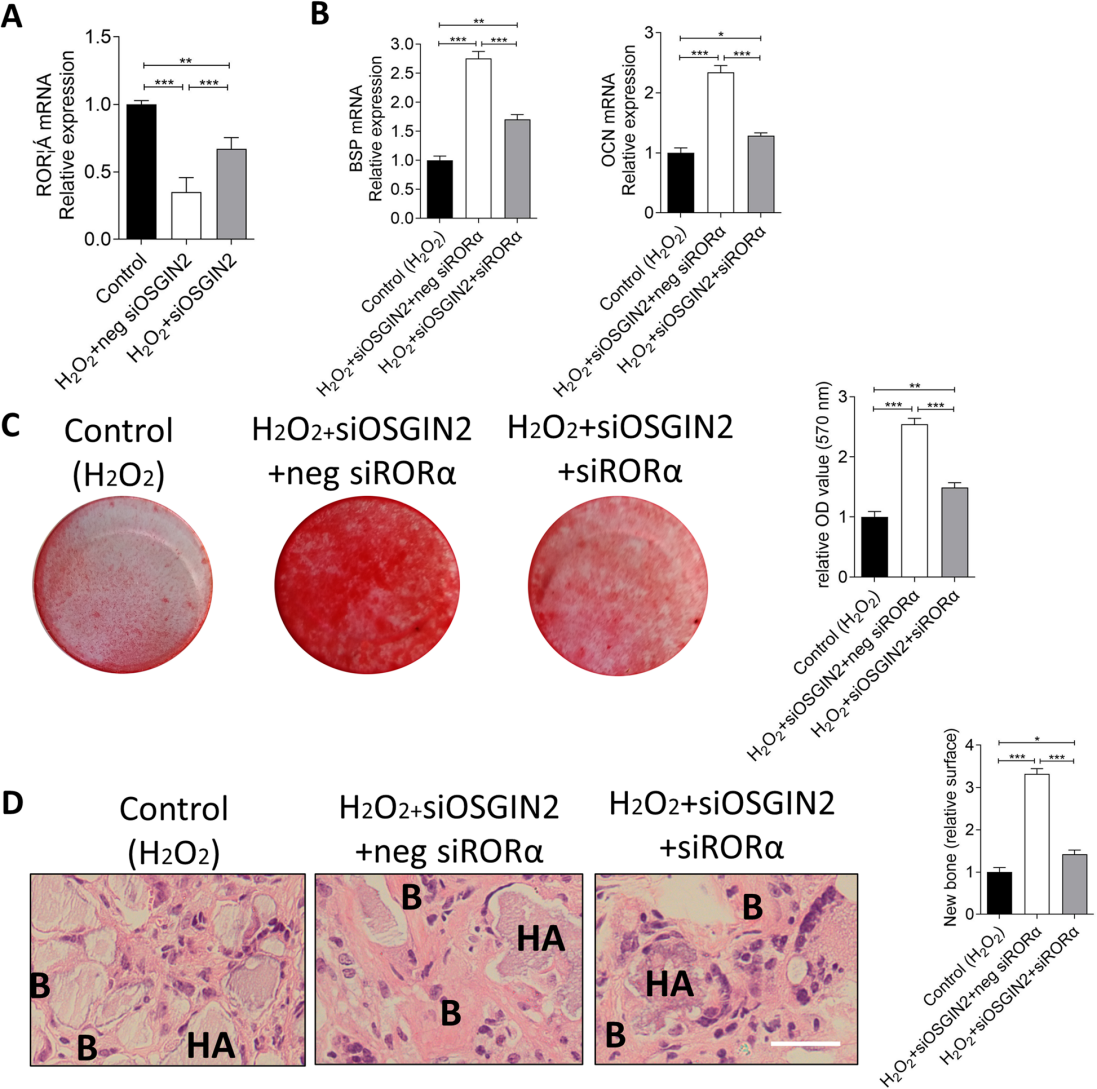
OSGIN2 regulates jawbone BMSCs osteogenesis under oxidative stress via RORα/BSP-OCN signaling. **A** Expression of RORα mRNA was detected using qPCR after treatment of H_2_O_2_ and anti-OSGIN2 (Control as control group: no H_2_O_2_, siOSGIN2 or negative control of siOSGIN2 treatment). **B** Expressions of BSP and OCN mRNAs were detected using qPCR, **C** Mineralized nodules were detected using alizarin red, **D** Ectopic bone formations were detected using H&E after treatment of H_2_O_2_, anti-OSGIN2 and anti-RORα (Control (H_2_O_2_) as control group: H_2_O_2_ treatment, but no siOSGIN2, siRORα or negative control of siRORα treatment). *n* = 5. Scale bar: 500 μm. *p < 0.05, **p < 0.01, ***p < 0.001

RORα, an osteogenic related molecule, was decreased under oxidative stress, while inhibition of OSGIN2 partially rescued its expression (Fig. 4A). These indicated that OSGIN2 was the upstream signaling of RORα. To determine whether OSGIN2/RORα signaling participated in regulation of jawbone BMSCs osteogenesis under oxidative stress, gain and loss assays were conducted. The data showed that silence of RORα restricted the osteogenesis recovery triggered by OSGIN2 inhibition, such as reduction of BSP and OCN (Fig. 4B), compromised mineralized nodules (Fig. 4C) and subcutaneous new bone formation (Fig. 4D). These data implied that OSGIN2 inhibited jawbone BMSCs osteogenesis under oxidative stress via regulating RORα/BSP-OCN signaling.

### OSGIN2 is a target for treatment of osteoporotic jawbone

**Fig. 5:**
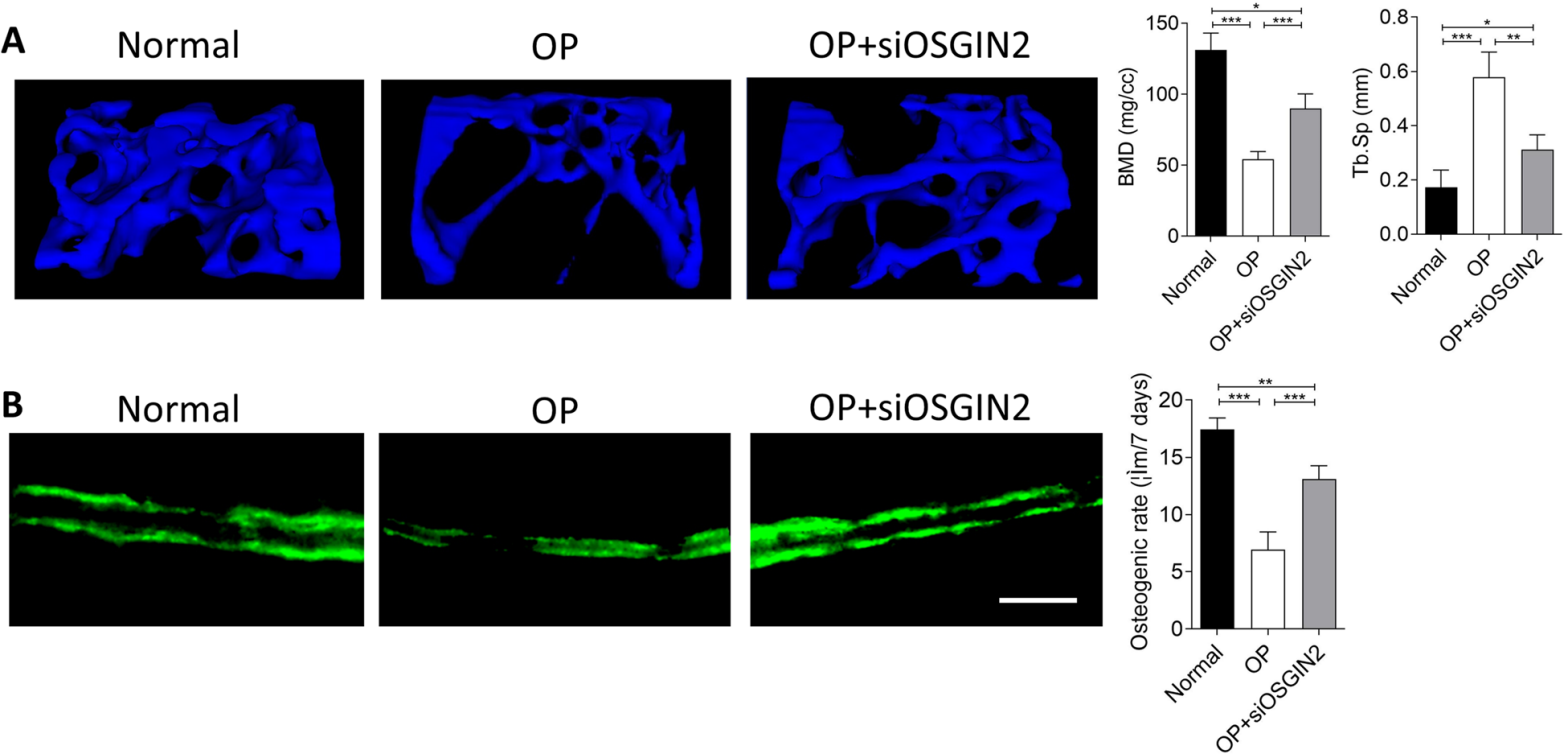
Effect of OSGIN2 regulation in treatment of osteoporotic jawbone. **A** trabecular bone and its parameters of jawbone were detected using microCT, **B** New bone deposition was detected using calcein labeling after intra-jawbone injection of siOSGIN2 (Normal as control group: non-osteoporosis and no treatment with siOSGIN2). *n* = 5. Scale bar: 500 μm. *p < 0.05, **p < 0.01, ***p < 0.001. OP: osteoporosis

To investigate the therapeutic role of OSGIN2 in the treatment of osteoporotic jawbone, siRNA against OSGIN2 was injected into jawbone of osteoporotic rats. The data exhibited that the jawbone mass of osteoporotic rats administrated with siRNA was notably increased, compared to osteoporotic rats with no treatment (Fig. 5A). Additionally, the bone parameters of bone mineral density and trabecular bone space were also ameliorated (Fig. 5A). Furthermore, calcein labeling assay showed that new bone deposition of osteoporotic rats was improved after treatment with siRNA against OSGIN2 (Fig. 5B). These results indicated that OSGIN2 was a target for treatment of osteoporotic jawbone.

## Discussion

Oxidative stress is featured by an augmentation of ROS that unbalances the intracellular redox system [28]. Recently, emerging studies have verified that ROS is a crucial regulator of bone cell function and that oxidative stress influences the pathophysiological process of skeletal system [29–31]. Previous studies have demonstrated the relationship between oxidative stress and osteoporosis [14]. Declined bone mineral density and deterioration of trabecular bone structure, as well as jawbone degeneration, are regarded to be associated with excessive oxidative stress in osteoporotic individuals [32–34]. Recent studies report that redundant ROS or oxidative stress triggers an overall restriction of osteoblastic differentiation of osteoblasts and BMSCs derived from long bones [35, 36]. However, there is a great difference between the developmental patterns of jawbone and long bone, meanwhile the jawbone BMSCs show different features from long bone BMSCs. It remains unknown whether oxidative stress affects jawbone BMSCs osteogenesis. In this study, we found that the ROS level was increased, while osteogenic markers decreased in osteoporotic jawbone BMSCs. Furthermore, *in-vitro* cellular model of oxidative stress also showed declined osteogenic markers (BSP and OCN) expressions. All these indicated that oxidative stress affected osteogenesis of jawbone BMSCs during osteoporosis.

OSGIN family, containing OSGIN1 and OSGIN2, is a type of highly reactive molecules of oxidative stress, which abundantly expresses in skeleton system (https://www.genecards.org/I). OSGIN1 has been reported to mediate the autophagy induced by oxidative stress to affect cell proliferation, apoptosis and differentiation [37, 38]. Additionally, OSGIN1 also involves in the regulation of apoptosis via ROS-PI3K/Akt/Nrf2 signaling [39]. However, OSGIN2 has been seldom investigated. According to GeneCards database, OSGIN2 is related to bone disorders accompanied with Nijmegen Breakage Syndrome, retinitis pigmentosa type 62, and so forth (https://www.genecards.org/I, http://www.malacards.org/card/retinitis_pigmentosa_62I). Our data demonstrated that OSGIN2 up-regulated in osteoporotic jawbone BMSCs, which showed higher ROS level compared to control. Furthermore, OSGIN2 is predicted to be associated with redundant oxidative stress to negatively regulate cell growth (https://www.genecards.org/I). Some studies reported that OSGIN2 sharply increased in dental-pulp-stem-cell-derived neurons under oxidative stress [40]. In addition, OSGIN2 is a key target gene of specific microRNA in cellular stress response [41]. OSGIN2 can also respond to the changes of peroxisome proliferator-activated receptor γ coactivator-1 (PGC-1)-related coactivator (PRC), which is involved in oxidative stress related biological process [41]. In this study, we found that OSGIN2 was positively related to ROS level, anti-OSGIN2 partially rescued the osteogenic capacity of BMSCs. All these implied the role of OSGIN2 in osteoporotic jawbone BMSCs fate regulation mediated by oxidative stress. However, the mechanism of OSGIN2 to influence bone metabolism remains unknown.

RORα regulates various metabolisms in different stem cells, tissues and organs, including bone and mesenchymal stem cells. After knocking-out RORα in mice, the growth and development of long bone are largely restricted, of which the main manifestations are thinning of long bone and bone loss [18]. BSP and OCN are well known as osteogenic markers that can improve the bone development and mineralization [20–23]. The role of BSP in skeletal mineralization is contributing the regulation on osteoblast and osteoclast balance, most prominently in primary/repair bone [21]. Overexpression of BSP promotes the mineralization, whereas absence of BSP negatively affects the local microenvironment of the bone tissue [21]. In addition, BSP also facilitates the osteogenic capacity of human BMSCs, which are the vital precursors of osteoblast for bone remodeling [24]. OCN has been investigated to modulate the balance of osteogenesis and adipogenesis of BMSCs, which is another crucial balance involved in bone metabolism [25]. Furthermore, OCN also alleviates osteogenic differentiation of BMSCs that is previously restricted under high glucose microenvironment [26]. All the researches indicates that BSP and OCN are significant osteogenic molecules involved in bone remodeling. Moreover, in the process of in-vitro differentiation, up-regulation of RORα expression initiates the transcription of osteogenic genes BSP and OCN, and then promotes the osteogenic differentiation of BMSCs [27]. This indicates that RORα plays an important role in bone metabolism. According to previous study, RORα can recruit synergistic factors such as PGC-1 to indirectly regulate cellular metabolism [18, 19]. Therefore, there might be some relevance between OSGIN2 and RORα. We found that anti-OSGIN2 remedied the H_2_O_2_-induced decline of RORα. Moreover, anti-RORα antagonized the protective effect of anti-OSGIN2 on BMSCs osteogenesis. All these suggested that RORα/BSP-OCN might be the downstream of OSGIN2 to modulate bone remodeling. Infusion of anti-OSGIN2 ameliorated osteoporotic jawbone loss and improved the bone formation, which verified the role of OSGIN2 in osteoporosis.

## Conclusions

**Fig. 6:**
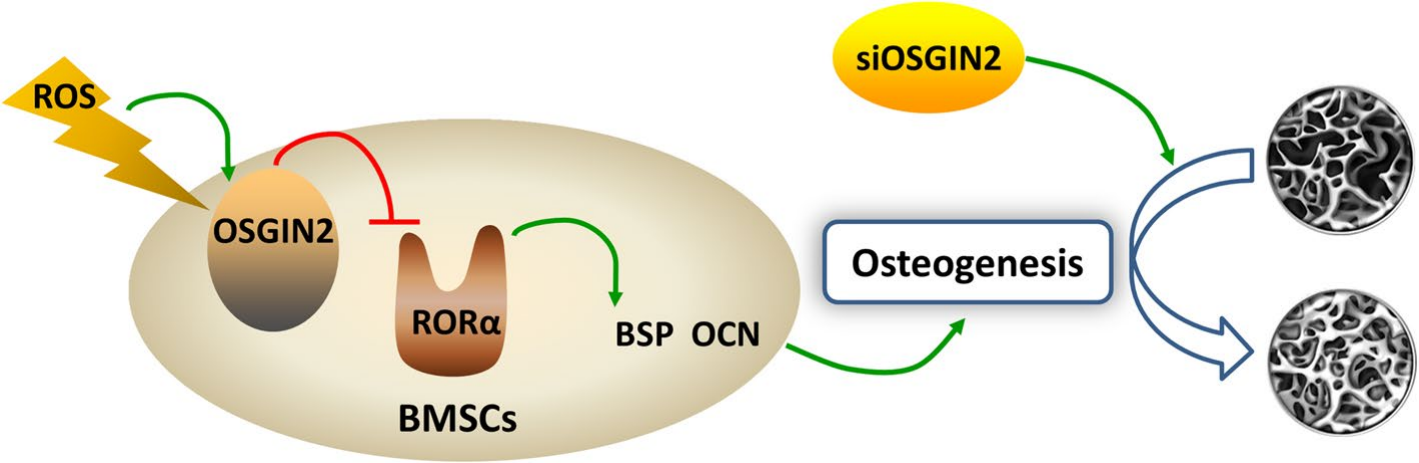
Schematic diagram of ROS/OSGIN2/RORα/BSP-OCN signaling in jawbone BMSCs during osteoporosis

Our study showed that oxidative stress was increased in jawbone BMSCs during osteoporosis. OSGIN2 prohibited osteogenesis of jawbone BMSCs via regulating RORα/BSP-OCN signaling under oxidative stress. OSGIN2 might be a novel target for osteoporotic jawbone loss (Fig. 6).

## Materials and methods

### Animals

All animals were acquired from the Department of Comparative Medicine of Jinling Hospital. Fifty-five 8-week-old female Sprague Dawley rats were used to establish osteoporosis model and to obtain BMSCs. Fourteen 8-week-old female nude mice were used to conduct ectopic bone formation assay. Animals of each group were raised in the same cage and surroundings (22 °C, 12 h light/12 h dark cycles, 50%–55% humidity) together. Each group contained five animals. All the conductions of compared groups were conducted at the same time interval. Random numbering method was used for grouping. Animals with death or sickness were excluded from the study. Pentobarbital sodium was used to kill pain of animals during the operations. All the animal experiments were performed in full compliance with the guidelines of National Institutes of Health and Animal Care Committee of Jinling Hospital, and the experimental protocols were approved by Ethics Review Board of Jinling Hospital (2019JLHKJDWLS-001) in this study. The study was carried out in compliance with the ARRIVE guidelines.

### BMSCs culture

BMSCs were flushed out from jawbone marrow of Sprague Dawley rats using precooled α-MEM medium (Gibco, USA) and were seeded in 10-cm dishes, which contained α-MEM medium, 10% fetal bovine serum, 1% streptomycin and penicillin (Gibco). BMSCs were cultured in the condition of 37 °C, 5% CO_2_ and saturation humidity. The medium was refreshed every two days. BMSCs were digested using 0.25% trypsin/1 mM EDTA (Gibco) until 75% confluence. BMSCs of passage 2 were applied for following experiments.

### Oxidative stress induction and antioxidant treatment

BMSCs were administrated with 100 μM H_2_O_2_ at 37℃ for one hour, while the controls were treated with phosphate buffered solution of equal quantity. Catalase (CAT) (Sigma-Aldrich, USA) was used as an antagonist to H_2_O_2_ with a concentration of 200 U/mL at 37℃.

### Reactive oxygen species measurement

For intracellular ROS estimation, BMSCs were labeled with 25 mM 2', 7'-dichlorofluorescein diacetate (DCFH-DA) (Beyotime, China) in serum-free medium and were incubated at 37℃ for 30 min. Then, BMSCs were rinsed and suspended in phosphate buffered solution, and were analyzed by flow cytometer (Beckman Coulter, USA) at 488 nm excitation and 525 nm emission wavelength. The ROS levels of adhesive BMSCs were tested using fluorescence microscope (Olympus Optical, Japan).

### RNA extraction and q-PCR analysis

Total RNAs of BMSCs were isolated with Trizol reagent (Invitrogen, USA) in accordance with the instructions. 500 ng total RNAs were used for reverse transcription to obtain cDNAs by a PrimeScript RT Reagent Kit (TaKaRa, Japan). SYBR Premix Ex Taq II kit (TaKaRa) was used for q-PCR analysis, and samples were amplified using a CFX96TM q-PCR System (Bio-Rad, USA). β-actin was used as the internal control. The forward and reverse primer sequences for q-PCR were displayed in Supplementary Table S1.

### Western blot analysis

BMSCs were lysed using lysis buffer containing protease inhibitor, and then were further lysed using low frequency ultrasound. The supernatant was collected for western blot assay. 15% SDS-PAGE was used to separate proteins with different molecular weights. Polyvinylidene fluoride membranes (Millipore, USA) were applied for proteins transfer, and were blocked using 5% non-fat milk powder buffer. Then the membranes were incubated at 4℃with the primary antibody for OSGIN2 (Antibodies-online, USA) and β-actin (Cell Signaling, USA) for 10 h. Subsequently, the membranes were rinsed and incubated with secondary antibody (Boster, China) for 2 h at room temperature. The membranes were developed for protein blot imaging. The blot gray values were analyzed with β-actin normalization using Photoshop CS7 (Adobe Systems, USA). The blots were cut priorly to hybridisation with antibodies during blotting.

### Alizarin red staining

BMSCs were seeded in 12-well plates with a quantity of 1 × 10^5^ per well. After 75% cellular confluence, osteogenic induction medium with 10 nM dexamethasone, 50 μg/mL ascorbic acid and 5 mM β-glycerophosphate (Sigma-Aldrich) was used to induce BMSCs osteogenic differentiation. After 28 days induction, alizarin red (Beyotime) was applied for staining of mineralized nodules. 6% cetyl-pyridine (Beyotime) was used to dissolve alizarin-red-positive mineralized nodules for quantification, and the solution was detected at 570 nm wavelength.

### Ectopic bone formation assay

BMSCs were cultured to construct cell sheets. A sandwich structure of three layers of BMSCs sheets composited with two layers of HA/TCP (20 mg, HA/TCP = 6/4, 50–200 nm) (Sigma-Aldrich) were packaged for subcutaneous grafting. Under anesthesia with 30 mg/kg pentobarbital sodium, the implants were transplanted on the back of 8-week-old NOD/SCID mice subcutaneously. Twelve weeks after transplantation, the implants were collected for fixing, decalcification and H&E staining. The areas of new bone formation were photographed and were estimated by Image-Pro Plus 6.0 (Media Cybernetics).

### BMSCs transfection

Duplex oligonucleotides of siRNAs against rat OSGIN2, RORα and their negative controls (Ribo Bio, China) were chemically modified with 2’-O-Methyl and transfected into the BMSCs at a working concentration of 100 nM using the siPORT NeoFX (Ambion, USA) according to the protocol.

### Injection of SiRNAs against OSGIN2

Four weeks after ovariectomy surgery, rats were administrated with siRNAs against rat OSGIN2 or its negative control (Ribo Bio) once a week for 4 weeks. In detail, the jawbones were exposed by surgery under anesthesia. 1 ng siRNAs against OSGIN2 or negative control were diluted in 25 μL sterile H_2_O for intra-jawbone injection using a micro-injector.

### Micro-CT analysis

The jawbones were scanned by micro-CT (GE, Fairfield, CT, USA) in accordance with the working protocol (voltage- 80 kV, current- 80 μA and isotropic resolution- 14 mm). Bone mass density (BMD) and trabecular space (Tb.Sp) were estimated by supporting analysis system (GE).

### Calcein labeling assay

Rats were given subcutaneous injection of calcein solution (8 mg/kg) (Sigma-Aldrich) at the 3^rd^ and 10^th^ day before their sacrifice. The jawbones were isolated, fixed and embedded. The embedded specimens of jawbones were cut into sections of 50 μm thickness, and then the calcein labeling was observed using a fluorescence microscope (Olympus). The distance between two adjacent green fluorescent bands was measured with Image Pro software to calculate new bone deposition rate of 7 days.

### Statistical analysis

All the data were showed as mean ± SD. Comparisons were tested by Student’s t-test or one-way ANOVA according to Normality test and Homogeneity test of variance using SPSS 13.0 (SPSS Inc, USA), otherwise, a nonparametric test would be used. Significant difference was determined at *p* < 0.05.

## Supplementary Information

**Additional file 1: Figure S1.** Cell surface markers of jawbone BMSCs. SCA-1, CD105, CD34 and CD45 were analyzed using flow cytometry. **Table S1.** Primers sequences.

## Data Availability

All data generated or analyzed during this study are included in this published article.
