# Supplementary material for: OSGIN2 regulates osteogenesis of jawbone BMSCs in osteoporotic rats
Source: BMC Mol Cell Biol. 2022 Jun 21;23:22. doi: 10.1186/s12860-022-00423-8 (PMC9215015; doi:10.1186/s12860-022-00423-8)

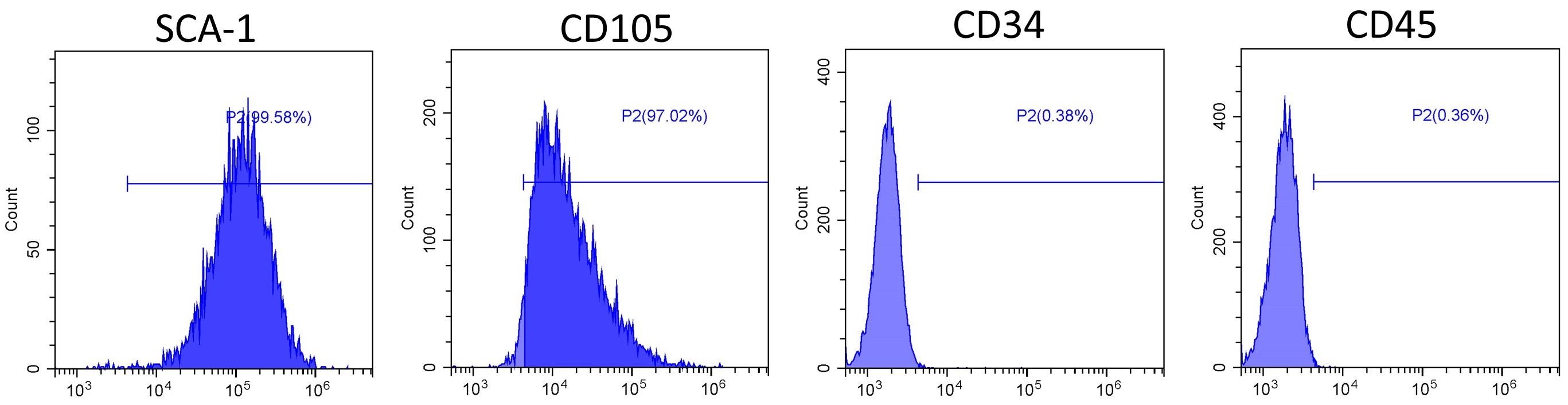


Fig. S1 Cell surface markers of jawbone BMSCs. SCA-1, CD105, CD34 and CD45 were analyzed using flow cytometry.

Table S1 Primers sequences

| Gene | Forward | Reverse |
| --- | --- | --- |
| *OSGIN2* | 5’- CAAGAGACAGCAGCATCA -3’ | 5’- TTCCTCCTATCCTACCTCAG-3’ |
| *BSP* | 5’- CACTCACTCACTTGCTCTC-3’ | 5’- GAACTATCGCCATCTCCATT-3’ |
| *OCN* | 5’- CCACCCGGGAGCAGTGT -3’ | 5’- GAGCTGCTGTGACATCCATACTTG -3’ |
| *RORα* | 5’- TCTGTCTGTCTGTCTGTCT-3’ | 5’- CACTGATAGGTAGGTCTTCC -3’ |
| *β-actin* | 5’- GGAGATTACTGCCCTGGCTCCTA -3’ | 5’- GACTCATCGTACTCCTGCTTGCTG -3’ |


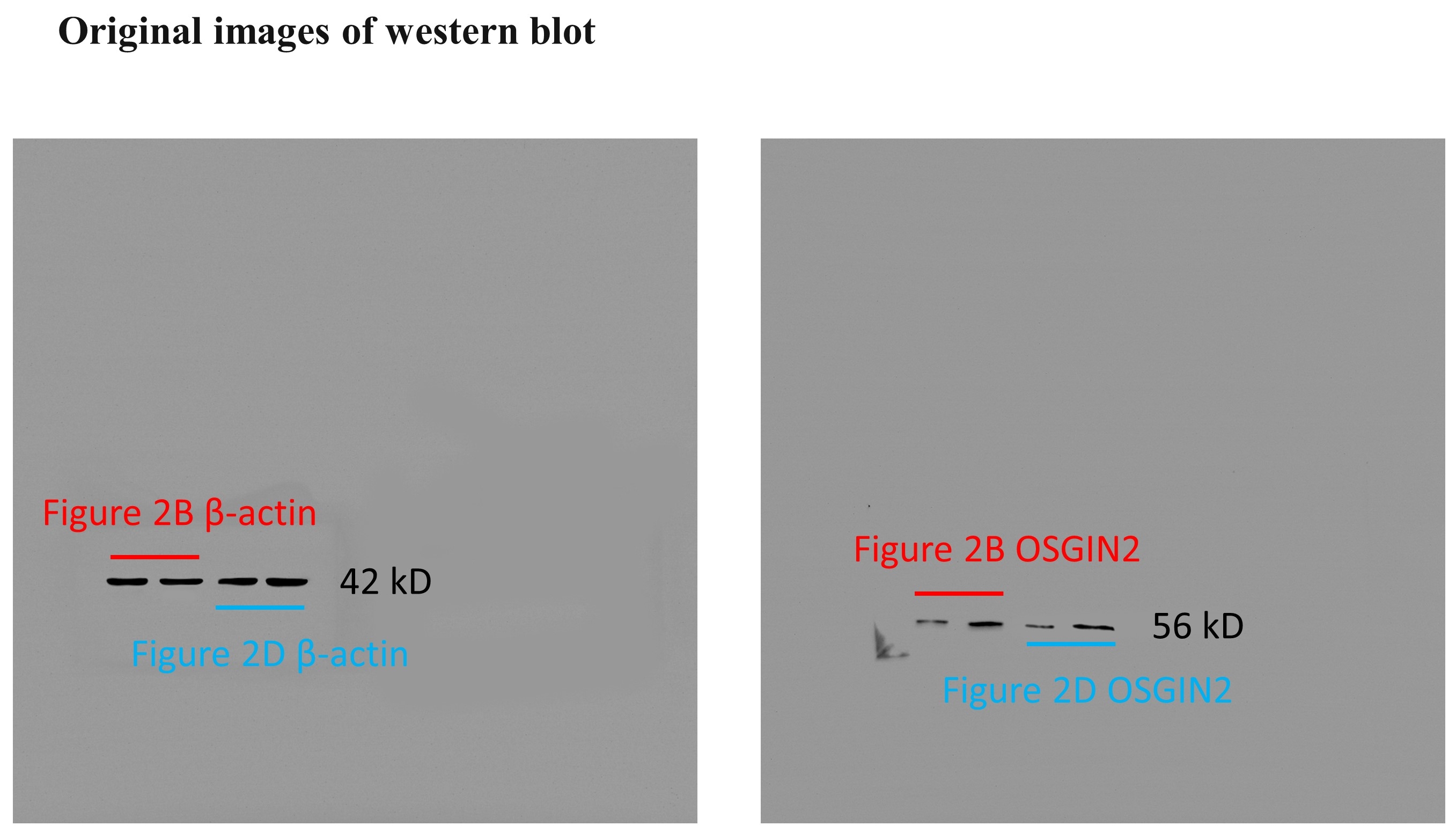

Supplement: Supplementary file 1 — Additional file 1: Figure S1. Cell surface markers of jawbone BMSCs. SCA-1, CD105, CD34 and CD45 were analyzed using flow cytometry. Table S1. Primers sequences. [file 12860_2022_423_MOESM1_ESM.docx]
